# Mesenchymal stem cells can prevent or promote the progression of colon cancer based on their timing of administration

**DOI:** 10.1186/s12967-023-04028-3

**Published:** 2023-03-28

**Authors:** Weiqian Hu, Weijun Wang, Xin Jiang, Zeyu Wang, Rong Lin

**Affiliations:** Department of Digestive, Union Hospital, Tongji Medical College, Huazhong University of Science and Technology, Wuhan, 430022 China

**Keywords:** Colitis-associated colon cancer, CD4^+^T cells, Immunomodulatory, Mesenchymal stem cells

## Abstract

**Background:**

Mesenchymal stem cell (MSC) therapy has been shown to have some therapeutic effects in rodent models and patients with IBD; however, its role in colon tumor models is controversial. In this study, the potential role and mechanisms of bone marrow-derived MSCs (BM-MSCs) in colitis-associated colon cancer (CAC) were investigated.

**Methods:**

The CAC mouse model was established with azoxymethane (AOM) and dextran sulfate sodium (DSS). The mice were administered an intraperitoneal injection of MSCs once weekly for different periods. The progression of CAC and the cytokine expression in tissues was assessed. Immunofluorescence staining was used to detect MSCs localization. Levels of immune cells in the spleen and lamina propria of the colon were detected using flow cytometry. A co-culture of MSCs and naïve T cells was performed to determine the effect of MSCs on naïve T cell differentiation.

**Results:**

Early administration of MSCs inhibited the occurrence of CAC, while late administration promoted the progression of CAC. The inhibitory effect of early injection in mice was characterized by the expression of inflammatory cytokines in colon tissue was decreased, and induction of T regulatory cells (Tregs) infiltration via TGF-β. The promotive effect of late injection was characterized by a shift of T helper (Th) 1/Th2 immune balance toward a Th2 phenotype through IL-4 secretion. IL-12 can reverse this shift to Th2 accumulation in mice.

**Conclusion:**

MSCs can curb the progression of colon cancer by inducing Treg accumulation via TGF-β at the early stage of inflammatory transformation but promote the progression of colon cancer by inducing a shift in Th1/Th2 immune balance to Th2 through IL-4 secretion at the late stage. And the immune balance of Th1/Th2 influenced by MSCs could be reversed by IL-12.

**Supplementary Information:**

The online version contains supplementary material available at 10.1186/s12967-023-04028-3.

## Background

Globally, colorectal cancer ranks third in terms of cancer causes and second in terms of cancer deaths [1]. It is increasingly clear that the tumor microenvironment, coordinated primarily by inflammatory cells, is an essential player in tumorigenesis, including cancer cell proliferation, survival, and migration. This study seeks to provide novel insights into the pathogenesis and therapeutic targets of colon cancer.

A complex relationship exists between T cell immune responses and colitis-associated colon cancer (CAC). In colorectal cancer, the type and location of immune cells are associated with clinical outcomes and survival [2]. T helper 1 (Th1) cells primarily secrete cytokines, such as interferon (IFN)-γ and tumor necrosis factor (TNF)-α, which can enhance the body’s autoimmune response and are important anti-cancer therapeutic agents [3]. Th2 cells, in contrast, secrete signature cytokines such as interleukin (IL)-4 and IL-10 [4], which coordinate humoral immunity [5] and promote allergic inflammatory responses [6]. Both Th1 and Th2 cells can secrete cytokines to promote their proliferation and inhibit each other’s proliferation and are in relative balance under normal circumstances [7]. However, when functional abnormalities occur, this balance is disrupted, known as “Th1/Th2 drift”, leading to a dominant Th1 or Th2 phenotype. In many tumors, the Th1/Th2 balance, with Th2 often dominating, may be related to immune escape [8]. The Th17 signature cytokine, IL-17A, may cause severe inflammation and affect autoimmune responses [9]. The expression of IL-17 in tumors can promote angiogenesis and tumor growth by elevating multiple pro-angiogenic factors and increasing the pro-inflammatory cytokines produced by tumor cells [10]. Chronic exposure to low levels of TH17-related cytokines may also contribute to cancer progression. T regulatory cells (Tregs) express the FoxP3 transcription factor and play a critical role in maintaining immune homeostasis and autoimmune disease. It is becoming increasingly clear that tumor-derived factors play a role in recruiting and amplifying FoxP3^+^Tregs, and the presence of FoxP3^+^Tregs in these tumors blocks effective immunity to cancer [11]. In addition, the tumor microenvironment (TME) may promote Treg activation, thereby suppressing anti-tumor responses. Therefore, Tregs not only inhibit intestinal inflammation but also improve the survival rate of tumor cells due to excessive immunosuppression. In summary, the function of T cells is influenced by several factors such as antigens, presence of co-stimuli, regulatory T cells, metabolic pathways, and soluble factors in the tumor microenvironment [3].

MSCs are pluripotent stem cells with significant immunomodulatory properties, long-term self-renewal, and multidirectional differentiation. They migrate toward inflammatory or neoplastic sites. In animals with colitis and inflammatory bowel disease (IBD), intravenous [12, 13] or intraperitoneal [14] injections of MSCs can considerably reduce colon inflammation, but timing is critical. Kawata et al. found that early injection of MSCs in a mouse model of DSS-induced colitis, increased anti-inflammatory cytokines and decreased pro-inflammatory cytokines; no such changes were observed when MSCs were injected at a later stage [15]. Conflicting results have been observed regarding the impact of MSCs therapy on colon tumor development. Co-injection of tumor cells with MSCs enhanced tumor development, liver metastasis, angiogenesis, and triggered tumor epithelial mesenchymal transition [16–19, 22]. However, some studies suggest otherwise. In azoxymethane/dextran sulfate sodium (AOM/DSS), MSCs were observed to reduce tumor formation [25–27]. Chen et al. reported that MSCs decreased tumor numbers through IL-6-STAT3 signaling [24]. We found that the tumor-promoting effect of MSCs was mostly observed in studies conducted on immunodeficient mouse models. Currently, the reasons for the different effects of MSCs on tumor growth are not clear; however, the effects may be influenced by a variety of factors, such as the mode and the timing of MSCs injection, and the tumor strain.

An essential component of the immunomodulatory activity of MSCs is their impact on T cells. MSCs can have different effects on T cells through the synergistic action of cell contact-dependent mechanisms [12] and soluble factors [13]. In addition, they can promote the formation of Tregs in vitro [14] and in vivo [15]. The protective impact of MSCs in Th1-mediated inflammatory and autoimmune diseases, such as type 1 diabetes mellitus [16] and Crohn’s disease [15], is correlated with the suppression of Th1 by MSC-induced Tregs. Similarly, Kavanagh et al. found that MSCs can also inhibit allergen-specific Th2 cell responses in allergic airway inflammation, in part by inducing Tregs [17]. Some studies [18, 19] suggest that MSCs might be more beneficial in shifting toward the Th2 phenotype. The Th17 cells have a pro-inflammatory effect, and MSCs may inhibit Th17 cells via IL-27 [20] or monocyte chemoattract protein-1 [21]; however, evidence of MSCs promoting the function of Th17 cells also exists [22]. The phenotypic plasticity potential of MSCs on CD4^+^T cell subsets has been associated with different in vivo conditions [2, 23].

In this study, we aimed to elucidate the role and mechanism of MSCs in altering tumor outcomes in an azoxymethane/dextran sulfate sodium (AOM/DSS) induced CAC model. Our findings can potentially pave the way for a change in targeted therapy directions.

## Methods

### Mice

Male C57BL/6 mice aged 6 to 8 weeks were purchased from Beijing Zolaibao Biotechnology Co., LTD. All mice were kept free of specific pathogens (SPF) for 12 h under light/dark cycles at a constant room temperature of 22 (± 2) °C and humidity of 55 (± 5)%. The mice received a continuous supply of food and water. Ethics approval was obtained from the Huazhong University of Science & Technology’s Tongji Medical College's Animal Care and Use Committee. The tenets of the Ministry of Health of China and the Helsinki Declaration were followed for all procedures (Document no. 55 of 2001).

### Animal treatment

Animals were fed for 1 week with no intervention and randomly divided into three groups (n = 45,15/group): (1) control group (CON) (2) early intervention with MSCs (2 × 10^6^ cells/0.2 mL per mouse; intraperitoneal injection) prior to first DSS feeding; AME group (3) late intervention with MSCs before the third DSS feeding; AML group [24]. At the start of week 0, 10 mg/kg of AOM was injected intraperitoneally (Sigma-Aldrich, St. Louis, MO, US). For a week, their drinking water contained DSS (2.5%, molecular mass 36–50 kDa; MP Biomedicals, Solon, OH, US), and regular water was provided for the following 2 weeks. Therefore, DSS was administered throughout the trial at weeks 1–2, 4–5, and 7–8. This led to the AOM and DSS-induced CAC mice models. MSCs [2 × 10^6^ cells/0.2 mL phosphate-buffered saline (PBS)] were administered to the AME group per week from week 1 and was administered to the AML group per week from week 7. Every week, mice in the CON group received an intraperitoneal injection of 0.2 mL of pure PBS. The weight of all subjects was measured and noted weekly. After the modeling was completed at week 10, the mice were fed for another 4 weeks and injected intraperitoneally pentobarbital sodium for euthanasia at week 14.

### MSCs isolation and culture

MSCs were isolated from the femur cavity of 4-week-old male C57BL/6 mice under aseptic conditions [25]. The cells were then cultured in a low-glucose DMEM medium with 10% fetal bovine serum (Gibco, NY, USA), cultured in a humidified atmosphere containing 5% CO_2_ at 37 °C, and unattached hematopoietic cells were removed by changing the medium. MSCs at passage 6 to 10 were used for the following experiments [26].

### H&E staining

Colon specimens were processed according to normal protocols, including immersion in 4% paraformaldehyde for 24 h, embedding, and dehydration [27]; 5 μm slices were stained with H&E, viewed, observed, and captured on camera using a light microscope.

### Immunohistochemistry

Deparaffinized paraffin-embedded sections (5 μm) were then rehydrated using a series of graded alcohol. After that, antigen heat retrieval was completed in citrate buffer using a pressure cooker, cooled to ambient temperature, and then blocked with a hydrogen peroxide solution. Antibodies (Abclone) for IFN-γ, TNF-α, TGF-β1, IL-4, IL-6, IL-10, IL-12, and IL-17 were added to the slides and incubated at 4 °C overnight. The slides were cleaned with PBS, then a secondary antibody conjugated to horseradish peroxidase was added, and incubated for 2 h. The slides were then treated with a DAB (3, 3′-diaminobenzidine) staining kit according to the manufacturer’s instructions for 10 min, and they were counterstained with hematoxylin for 2 min. A light microscope was used to take pictures of the sections.

### Immunofluorescence staining

We utilized donkey serum to block endogenous antigens. The paraffin-embedded sections (5 μm) were dewaxed and hydrated. Antigen retrieval was carried out in citrate buffer and then cooled to 26 °C temperature. At 4 °C a particular primary antibody directed against mouse GFP (Abcam, Cambridge, UK) was incubated with the tissue slices overnight. The slides were cleaned three times with PBS, incubated for 1 h with secondary antibodies from Antigen Biotech Co., Ltd., China, and stained for nuclei with 4′,6-diamidino-2-phenylindole dihydrochloride (DAPI). The samples were captured on camera using a fluorescence microscope (Olympus, Tokyo, Japan).

### Real-time quantitative PCR (RT-qPCR)

TRIzol reagent (Invitrogen, USA, 15596018) was used to extract total RNA, and a cDNA synthesis kit was used to transcribe the extracted total RNA into cDNA (Vazyme, China). qPCR was carried out using RT-PCR (Applied Biosystems) equipment. Additional file 1: Table S1 contains the list of the primer sequences used in this study.

### Western blot and protein isolation

Using RIPA Lysis Buffer (Beyotime, Jiangsu, China) supplemented with phenylmethyl sulfonyl fluoride (PMSF), a protease and phosphatase inhibitor, we extracted proteins from cells and colon tissues. Pierce^™^ BCA Protein Assay Kit (Thermo Fisher, US) was used to measure the total protein concentration. Denatured protein samples of the appropriate quality were then run through sodium dodecyl sulfate-polyacrylamide gel electrophoresis (SDS-PAGE) before being transferred to PVDF membranes. The membrane was incubated for 0.5 h in Tris-buffered saline with 0.1% Tween^®^ 20 Detergent containing 5% bovine serum albumin. After that, primary antibodies against β-actin, IFN-γ, TNF-α, and TGF-β1, IL-4, IL-6, IL-10, IL-12, and IL-17 were added to the membrane and incubated at 4 °C overnight. Alpha Innotech Corporation St. Leonardo' FluorChem FC2 technology was used to quantify the bands.

### Isolation of splenocytes and lamina propria lymphocytes (LPL)

Spleens were minced to prepare splenocytes. After that, splenocytes were washed with PBS containing EDTA and fetal calf serum (FCS), filtered through a 100 μm cell strainer, and rinsed with an erythrocyte lysis buffer. LPL were isolated as previously described [28]. Colons were cut open lengthwise, cleaned with PBS, and then cut into 1 cm pieces. Two PBS washes with EDTA were performed on tissue sections for 10 min at 37 °C while rotating the solution. After that, colon sections were washed twice with RPMI-1640 containing 1% FCS for 15 min at 37 °C while being rotated. Colon pieces were then thoroughly vortexed, cleaned with PBS, and digested for 90 min at 37 °C in RPMI-1640 with 20% FCS and 100 U/ml collagenase (Sigma-Aldrich).

### Co-culture and treatment

The spleen of the mouse was ground. Naïve T cells were inoculated into a 12-well culture plate containing RPMI-1640 (Gibco, Grand Island, US), 10% fetal bovine serum, and anti-CD3/CD28 monoclonal antibodies (EBioscience, San Diego, CA, USA). Then, using a 3 μm polycarbonate filter, MSCs were transferred to the top chamber of a 12-well Transwell cell and cultivated alongside naïve T cells in an incubator at 37 °C with 5%CO_2_. To examine the impact of MSCs on the differentiation of naïve T cells, TGF-β1, IL-4, and IL-12 were added separately (Miltenyi Biotec Bergisch Gladbach, Germany) to the chamber. In general, we had three groups: CD4^+^T cells, CD4^+^T cells cultivated with stimulating factor, and CD4^+^T cells cultured with MSCs. Cells from the chamber were collected and analyzed.

### Statistical analysis

Data are represented as the mean and standard error of the mean. For statistical analysis and mapping, GraphPad Prism software (version 8.0) and SPSS software (version 22.0) were used. A *P*-value < 0.05 was considered statistically significant.

## Results

### MSCs migrate to the colon and influence the development of AOM/DSS-induced colon tumors

**Fig. 1:**
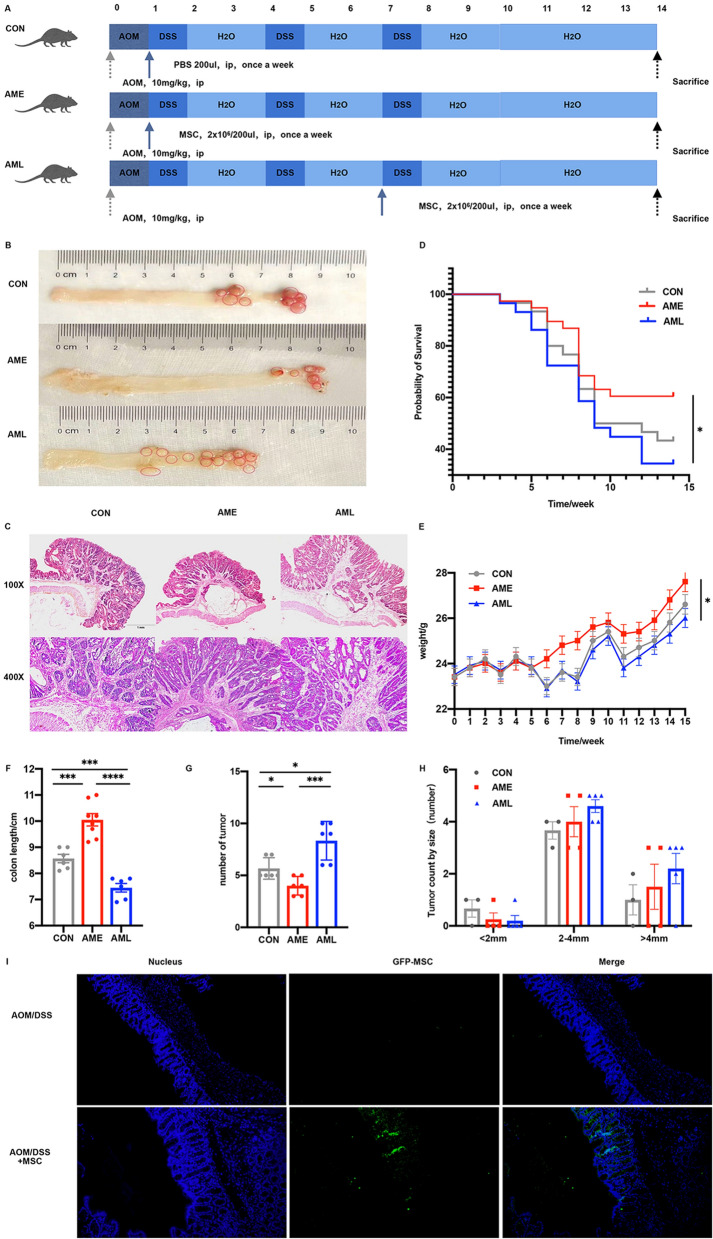
MSCs affect the growth of colon cancers brought on by AOM/DSS. **A** After AOM injection, animals were subjected to three cycles of DSS. MSCs during the study period, the AME group was injected (2 × 10^6^ cells/mouse) per week from week 1, while the AML group was injected (2 × 10^6^ cells/mouse) per week from week 7. Every week, mice in the CON group received an intraperitoneal injection of 0.2 mL of pure PBS. Animals were sacrificed under anesthesia at week 14 (n = 15/group). **B** Colons were excised for macroscopic observation and **C** slices were stained with H&E observed using the light microscope. **D** The survival rate and **E** weight of mice in each group was noted and compared. **F** Colon length, **G** tumor numbers, and **H** tumor numbers counted by size were assessed. **I** Colon tissues were collected to detect the migration of MSCs in the DSS/AOM-induced model. Data are shown as mean ± SD from three independent experiments. ***P < 0.001, **P < 0.01, *P < 0.05

To determine their therapeutic effects, MSCs were used to treat AOM/DSS-induced CAC (Fig. 1A). Significant weight loss and bloody diarrhea were seen in the AOM/DSS-treated mice. Typically, tumors were found between the middle of the colon and the distal rectum (Fig. 1B). This region corresponds to the main site of human colorectal cancer. H&E staining of the colon tissue revealed crypt epithelial deformation, extensive mucosal damage, and inflammatory cell infiltration in all three groups (Fig. 1C). Compared with the AML group, the mice in the AME group had a better survival rate (Fig. 1D; *P* < 0.05). Compared with the CON group, the AME and AML groups presented with less and more weight loss, respectively (Fig. 1E; *P* < 0.05). Compared with the CON group, the colons of the mice in the AML group were significantly shorter. In contrast, the AME group significantly alleviated the AOM/DSS-induced colon shortening (Fig. 1F; *P* < 0.01). There were significantly fewer colonic tumors in the AME group and more tumors in the AML group compared to the CON group (Fig. 1G; *P* < 0.05). There were no significant differences among the groups concerning the tumor sizes (Fig. 1H). No MSCs were detected in the colon tissue of the mice in the CON group; however, they were detected in the colon tissue of the group treated with MSCs (i.e., the AME and AML groups) (Fig. 1I). Hence, MSCs can migrate to the colon tissue in response to intestinal injury.

### MSCs induce changes in T cell immune balance in mice

**Fig. 2:**
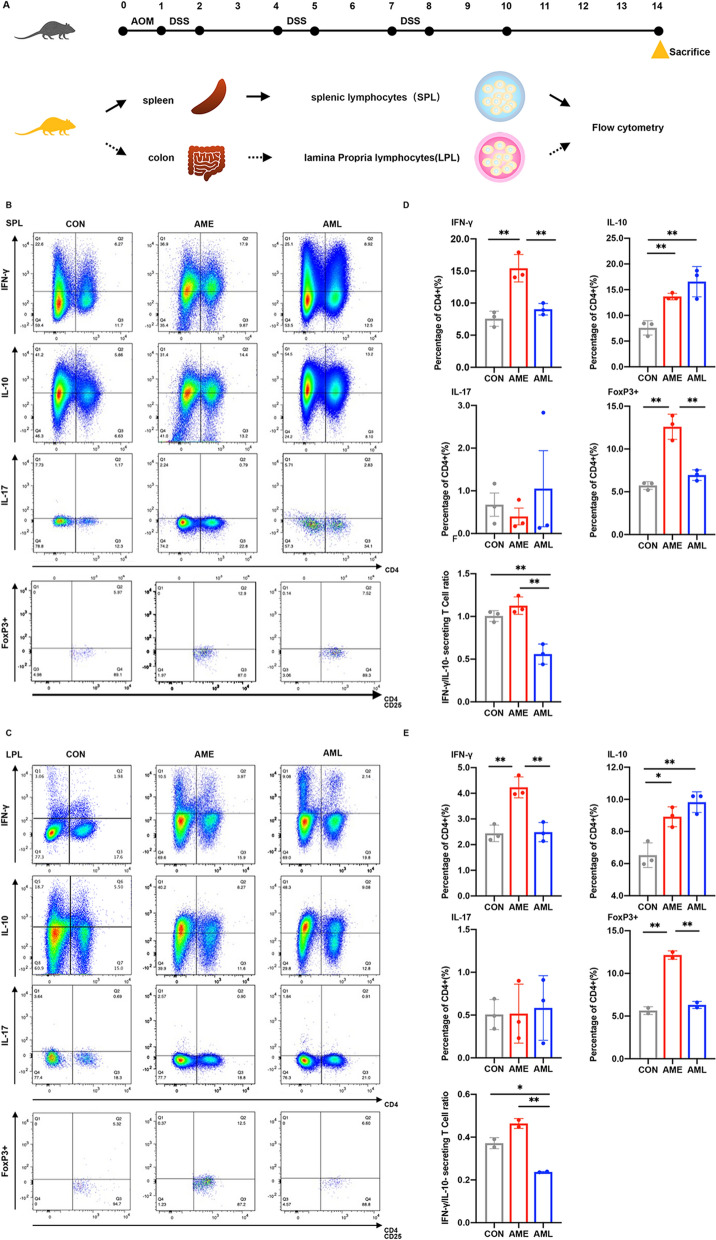
MSCs induce changes in T cell immune balance in mice. **A** After AOM injection, animals were subjected to three cycles of DSS/H_2_O. Animals were sacrificed under anesthesia at week 14.And isolating SPL and LPL, flow cytometry was performed separately. **B** Flow cytometry analysis of the mean percentage of Th1, Th2, Th17 and Treg cells in splenic. **C** Flow cytometry analyzed the mean percentage of Th1, Th2, Th17 and Treg in lymphocyte of lamina propria of colon. **D** analyze and count the mean percentage of Th1, Th2, Th17 and Treg cells in splenic. And **E** analyze and count the mean percentage of Th1, Th2, Th17 and Treg cells in lymphocyte of lamina propria of colon. *SPL* splenocytes, *LPL* lamina propria lymphocytes. Values are expressed as means ± SD.*P < 0.05, or **P < 0.01

T cell infiltration was analyzed by flow cytometry in AOM/DSS-induced colon cancer (Fig. 2A). Compared with that in the CON group, the proportion of CD4^+^CD25^+^Foxp3^+^ Tregs in splenic tissues (Fig. 2B) and lymphocytes of lamina propria of the colon (Fig. 2C) of mice in the AME group was significantly increased by more than 2-fold (Fig. 2D, E; *P* < 0.01), while the immune balance shifted toward the Th2 phenotype in the AML group (Fig. 2D, E; *P* < 0.01). These findings suggest that early MSCs intervention inhibits the progression of CAC, possibly by inducing the accumulation of Tregs. The effect of late MSCs administration on cancer progression is more likely to be mediated by changes in the immune balance of Th1/Th2.

### MSCs regulate colon inflammation associated with the development of CAC

**Fig. 3:**
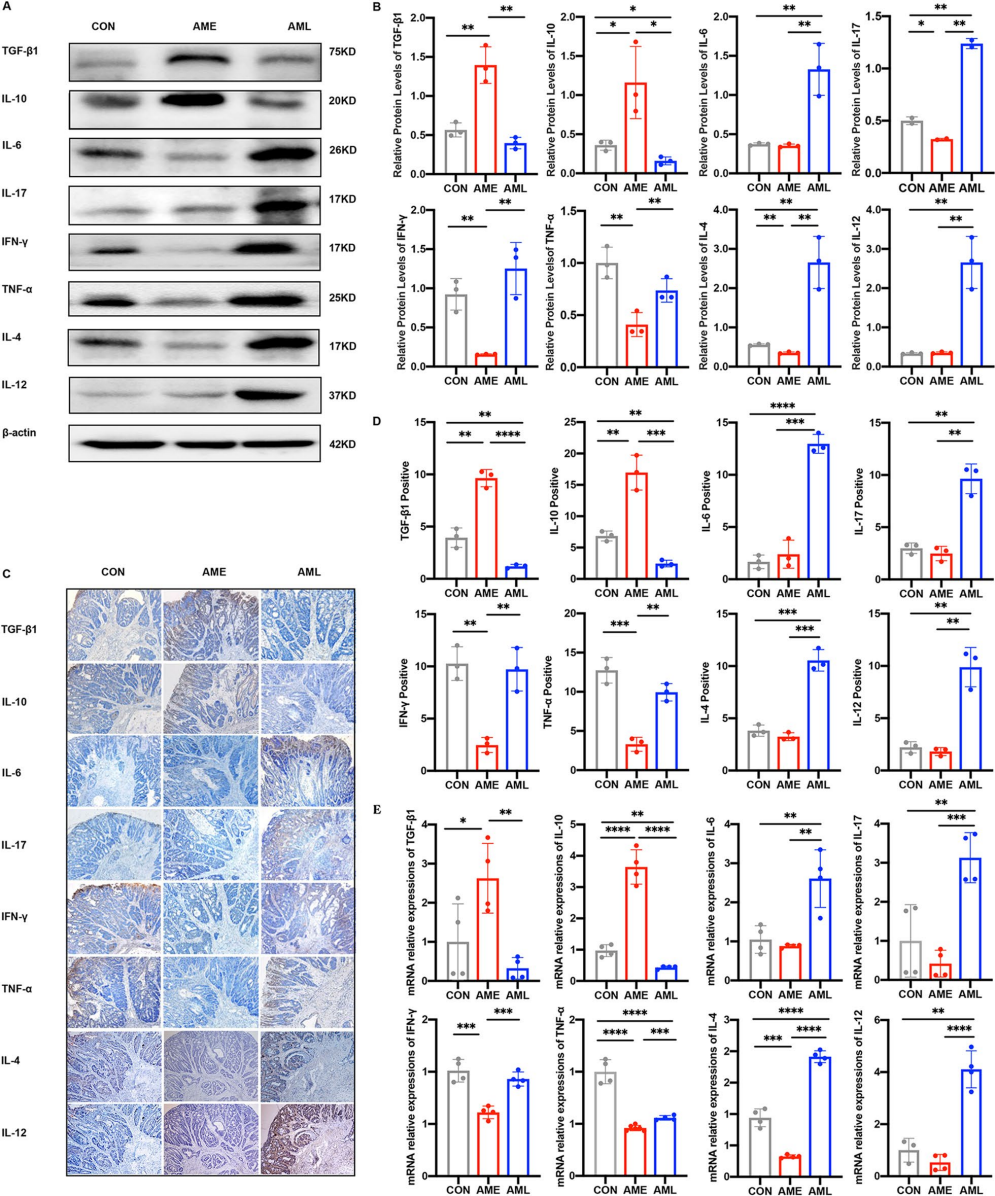
MSCs regulate colon inflammation associated with the development of CAC. We extracted colon tissues of mice. **A**, **B** Representative immunoblot bands and histogram of relative expression for the IFN-γ, TNF-α, TGF-β1 IL-4, IL-6, IL-10, IL-12 and IL-17 (n = 3). **C**, **D** Representative immunohistochemistry images and histogram of relative expression for the IFN-γ, TNF-α, TGF-β1 IL-4, IL-6, IL-10, IL-12 and IL-17 (n = 3). **E** The mRNA relative expressions of IFN-γ, TNF-α, TGF-β1 IL-4, IL-6, IL-10, IL-12 and IL-17 (n = 4). Values are expressed as means ± SD. *P < 0.05, **P < 0.01, or ***P < 0.001

We then assessed whether the effect of MSCs on colon tumors was related to their inflammatory properties. Major inflammatory factors related to T cell subsets were selected for comparison. Compared with those in the CON group, western blot (WB) analysis of protein expression levels of inflammatory cytokines (IL-4, IFN-γ, and TNF-α) in the AME group were decreased (Fig. 3A, B; *P* < 0.01), while those of the anti-inflammatory cytokines (TGF-β and IL-10) were significantly increased by twofold (Fig. 3A, B; *P* < 0.05. Full-length blots are presented in Additional file 2: Fig. S1). In the AML group, protein expression of inflammatory cytokines (IL-6, IL-4, IL-12, and IL-17A) increased by 2– 5-folds (Fig. 3A, B; *P* < 0.01), while anti-inflammatory cytokines (TGF-β and IL-10) decreased by more than half (Fig. 3A, B; *P* < 0.01). Similar phenomena were also observed by colon histological staining (Fig. 3C, D; *P* < 0.05) and mRNA expression (Fig. 3E; *P* < 0.05) in colon tissue. Taken together, these findings imply that, MSCs intervention at different time points may induce both pro-inflammatory and anti-inflammatory responses in AOM/DSS-induced CAC mice, depending on the initial time of MSCs administration.

### MSCs trigger Treg accumulation in mice via* TGF-β at an early stage*

**Fig. 4:**
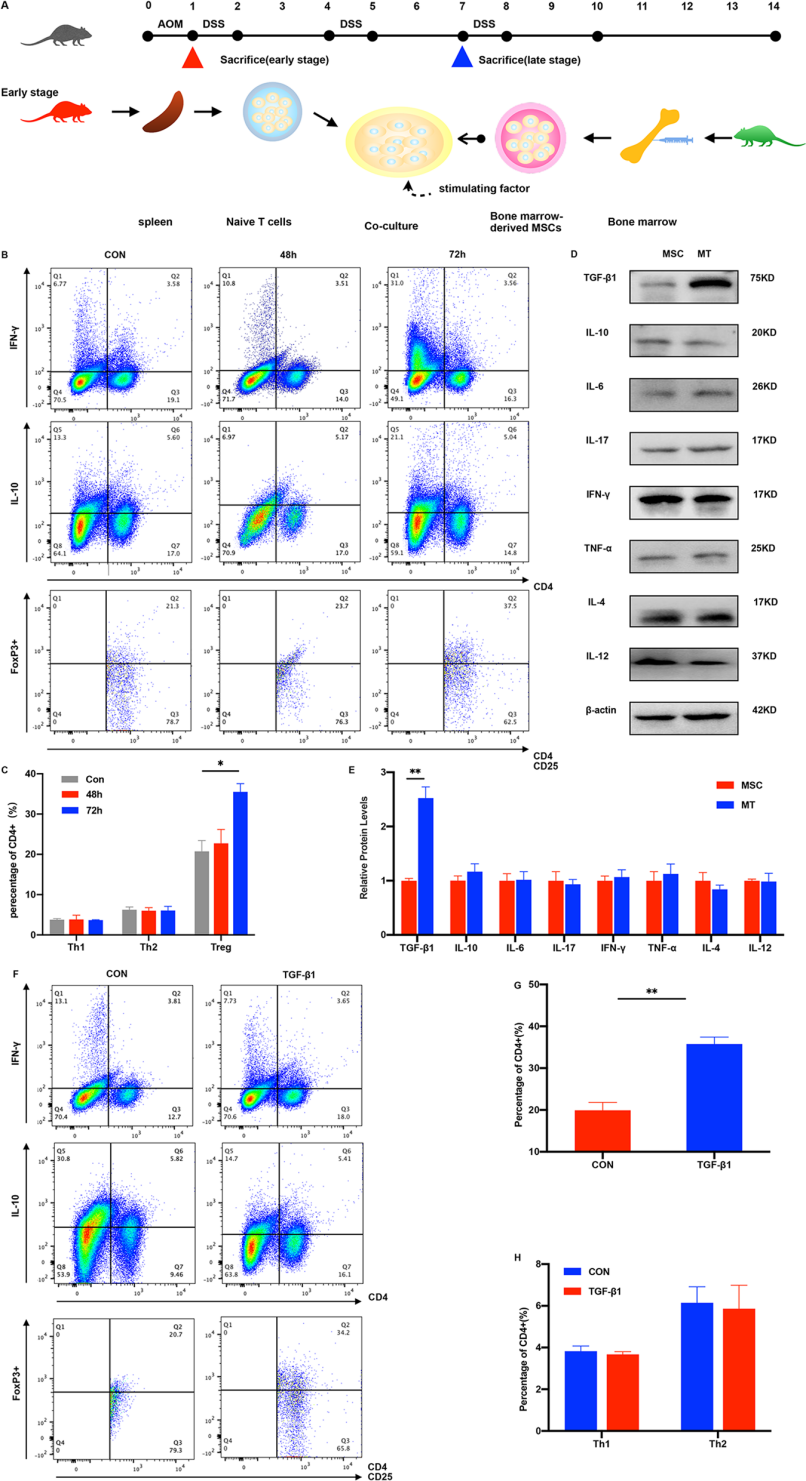
MSCs trigger Treg accumulation in mice via TGF-β at an early stage. **A** Scheme of the Co-culture and treatment experimental design. Some mice in the AME group were sacrificed 1 week after AOM application, and naïve primary T cells were extracted from the spleen of mice, and co-cultured with MSCs for 48 h and 72 h respectively. **B** Flow cytometry analysis of the mean percentage of Th1, Th2 and Treg cells in naïve CD4T cells co-cultured with MSCs (ratio 1:5) for 48 h and 72 h respectively or without MSCs. **C** Analyze and count separately. We extracted proteins from MSCs co-cultured for 72 h with naïve CD4^+^T. **D**, **E** Representative immunoblot bands and histogram of relative expression for the IFN-γ, TNF-α, TGF-β1 IL-4, IL-6, IL-10, IL-12 and IL-17. **F** Flow cytometry analysis of the mean percentage of Th1, Th2 and Treg cells in naïve CD4^+^T cells co-cultured with TGF-β (10 ng/ml) or without TGF-β. **G**, **H** Analyze and count separately. Values are expressed as means ± SD.*P < 0.05, **P < 0.01. *MSC* mesenchymal stem cells, *MT* MSCs co-cultured for 72 h with naïve CD4^+^T

To explore how MSCs regulate Tregs, mice in the AME group were sacrificed 1 week after AOM application, and naïve primary T cells were extracted from the spleen of mice and co-cultured with MSCs (Fig. 4A). After 72 h, the proportion of CD4^+^CD25^+^Foxp3^+^Tregs in the MSCs co-culture group was about 1.5-fold higher than that in the CON group alone (Fig. 4B, C; *P* < 0.05). However, this phenomenon was not distinctly higher in the co-cultured group only lasting 48 h (Fig. 4B, C). We extracted the cell protein of MSCs and after WB analysis found that the expression of TGF- β in MSCs co-cultured with naïve primary T cells was higher than that of MSCs alone (Fig. 4D). TGF-β increased 2.5-fold, while the other indicators showed no significant change (Fig. 4E; *P* < 0.01. Full-length blots are presented in Additional file 2: Fig. S2). For a further demonstration that the induction of MSCs is mediated by TGF-β, we added a TGF-β stimulator to the T cell culture medium. After 72 h, the percentage of CD4^+^CD25^+^Foxp3^+^Tregs in the group with a TGF-β stimulator increased more than 1.5-fold compared to the group without the TGF-β stimulator (CON) (Fig. 4F, G; *P* < 0.01). The Th1 and Th2 ratios also did not change significantly (Fig. 4C, H).

### MSCs skew Th1/Th2 immune balance toward Th2 via IL-4 at an advanced stage

**Fig. 5:**
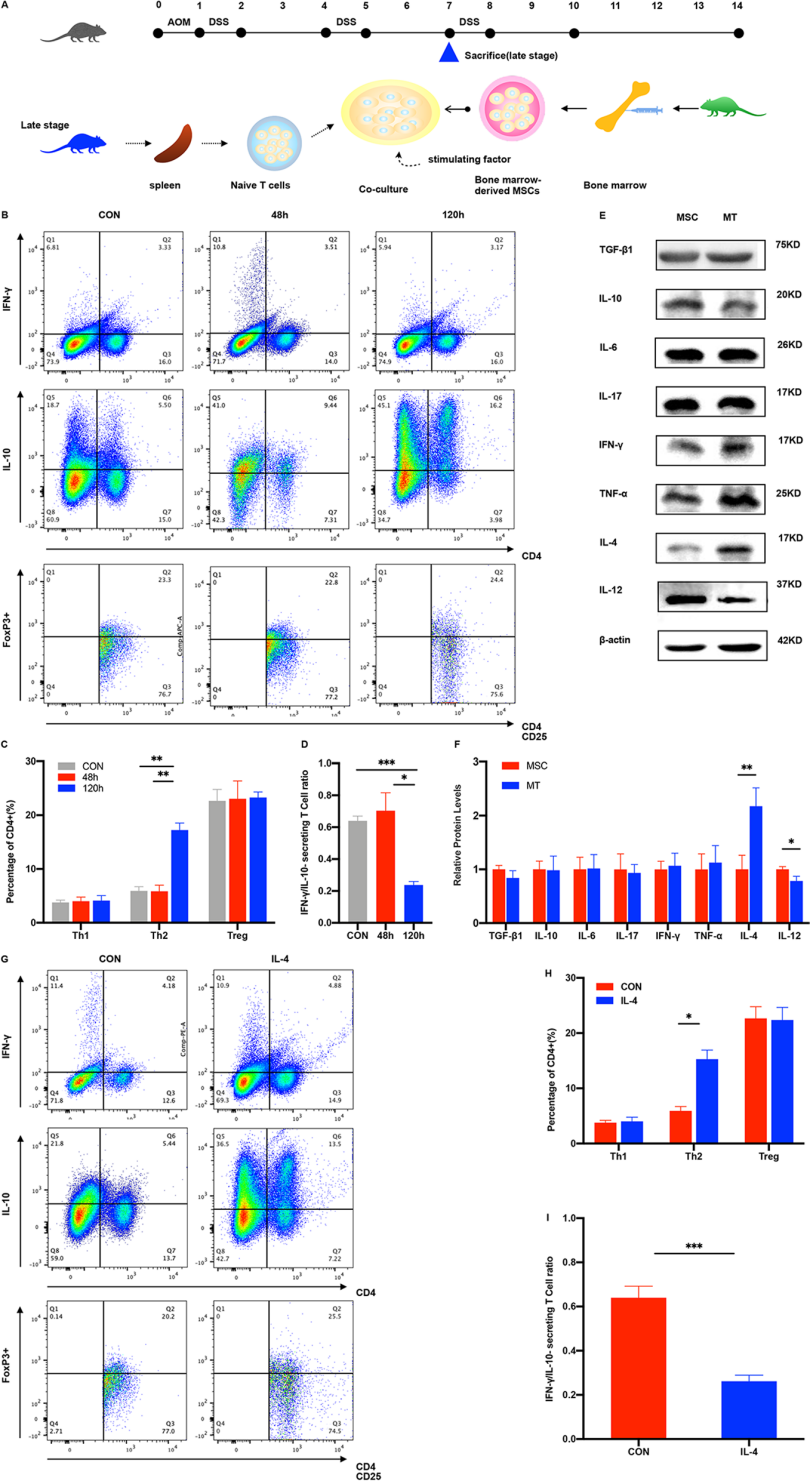
MSCs skew Th1/Th2 immune balance toward Th2 via IL-4 at an advanced stage. **A** Scheme of the Co-culture and treatment experimental design. Some mice in the AML group were sacrificed 7 weeks after AOM administration, and naïve primary T cells were extracted from the spleen of mice, and co-cultured with MSCs for 48 h and 120 h respectively. **B** Flow cytometry analysis of the mean percentage of Th1, Th2 and Treg cells in naïve CD4^+^T cells co-cultured with MSCs (ratio 1:5) for 48 h and 120 h respectively or without MSCs. **C**, **D** analyze and count separately. We extracted proteins from MSCs co-cultured for 120 h with naïve CD4^+^T. **E**, **F** Representative immunoblot bands and histogram of relative expression for the IFN-γ, TNF-α,TGF-β1 IL-4, IL-6, IL-10, IL-12 and IL-17. **G** Flow cytometry analysis of the mean percentage of Th1, Th2 and Treg cells in naïve CD4^+^T cells co-cultured with IL-4 (10 ng/ml) or without IL-4. **H**, **I** Analyze and count separately. Values are expressed as means ± SD. *P < 0.05, **P < 0.01 or ***P < 0.001. *MSC* mesenchymal stem cells, *MT* MSCs co-cultured for 120 h with naïve CD4^+^T

Similarly, to explore how MSCs regulate the balance of immune subtypes of Th cells, the mice in the AML group were sacrificed 7 weeks after AOM administration, and primary naïve T cells were extracted from the spleen and co-cultured with MSCs (Fig. 5A). After 120 h, the ratio of Th1/Th2 in T cells alone was about threefold higher than that in the MSCs co-culture group (Fig. 5B–D; *P* < 0.05). However, this phenomenon was not obvious in the co-cultured group only lasting 48 h (Fig. 5B–D). We extracted the cell protein of MSCs. Through WB analysis, it was found that compared with that of the MSCs alone, the expression of IL-4 of MSCs co-cultured with naïve primary T cells increased about twofold (Fig. 5E, F; *P* < 0.01. Full-length blots are presented in Additional file 2: Fig. S3), and the expression of IL-12 decreased (Fig. 5E, F; *P* < 0.05), and that of other indicators showed no significant changes. To further prove that IL-4 is the key to MSCs regulation, we added IL-4 stimulant to the T cell culture medium. After 120 h, the ratio of Th1/Th2 in the primary T cell-only group was still over twofold higher than that in the IL-4 stimulated group (Fig. 5G–I; *P* < 0.01). There was no significant difference in Tregs between groups (Fig. 5B, C, G, H).

### IL-12 reverses the shift of Th1/Th2 immune balance to Th2 accumulation in mice

**Fig. 6:**
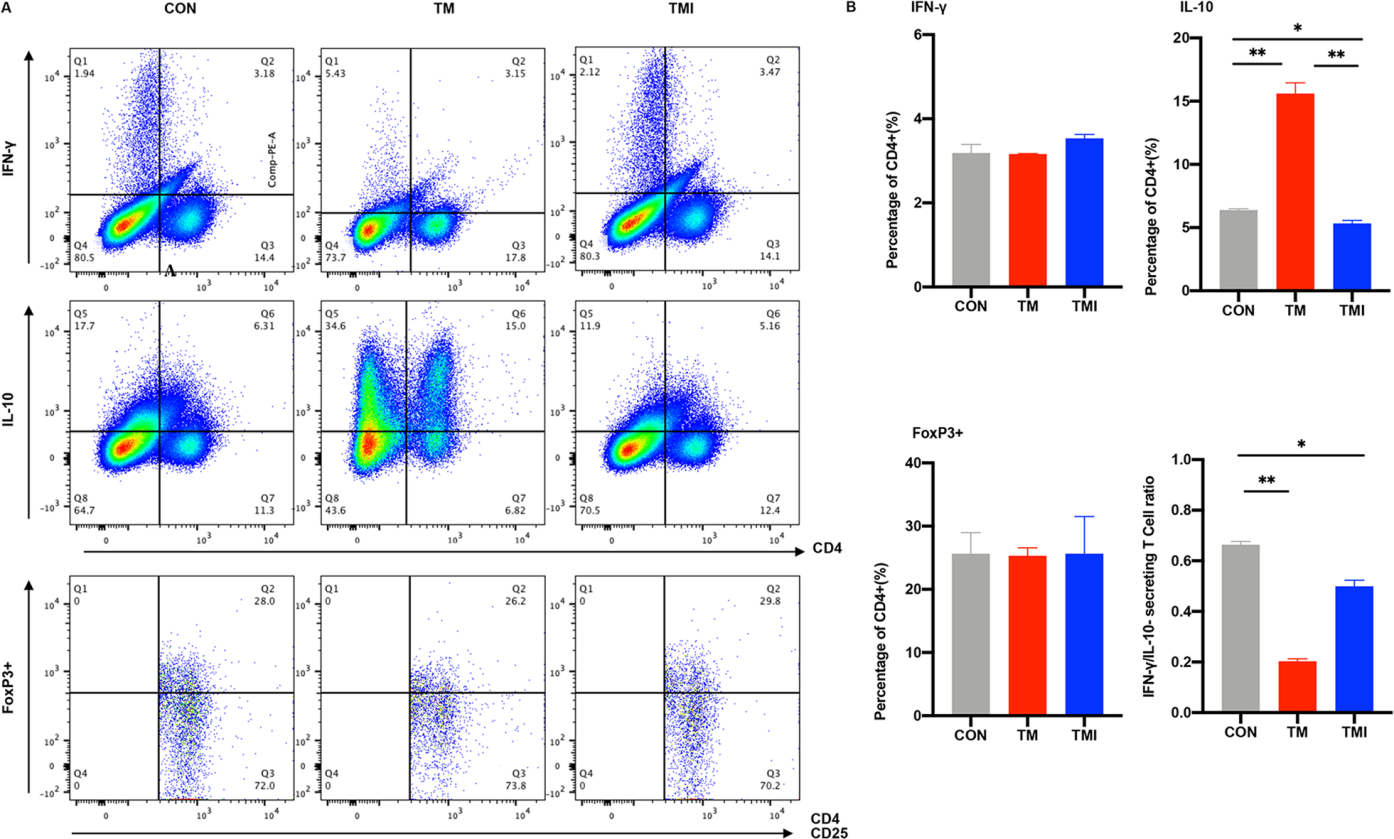
At an advanced stage, Il-12 reverses the shift of Th1/Th2 immune balance to Th2 accumulation. The mice in the AML group were sacrificed 7 weeks after AOM administration, and naïve primary T cells were extracted from the spleen of mice **A** Flow cytometry analysis of the mean percentage of Th1, Th2 and Treg cells in naïve CD4^+^T cells co-cultured with MSCs (ratio 1:5) or with MSCs/IL-12(10 ng/ml) for 72 h respectively. **B** Analyze and count separately. Values are expressed as means ± SD. *P < 0.05, **P < 0.01. TM: CD4^+^T cells co-cultured with MSCs for 72 h; TMI: CD4^+^T cells co-cultured with MSCs (ratio 1:5)/IL-12(10 ng/ml)

To further explore the effect of Th1/Th2 balance on tumors, mice in the AML group were sacrificed 7 weeks after AOM administration, and primary naïve T cells were extracted from the spleen of mice and co-cultured with MSCs. After 5 days, the Th1/Th2 ratio in the T cell-only group was approximately threefold higher than that in the MSCs co-culture group (Fig. 6A, B; *P* < 0.01). The proportion of Th1/Th2 increased when an IL-12 stimulator was added to the co-culture medium, suggesting that the immune balance of Th1/Th2 influenced by MSCs could be reversed by IL-12 (Fig. 6A, B; *P* < 0.05).

## Discussion

**Fig. 7:**
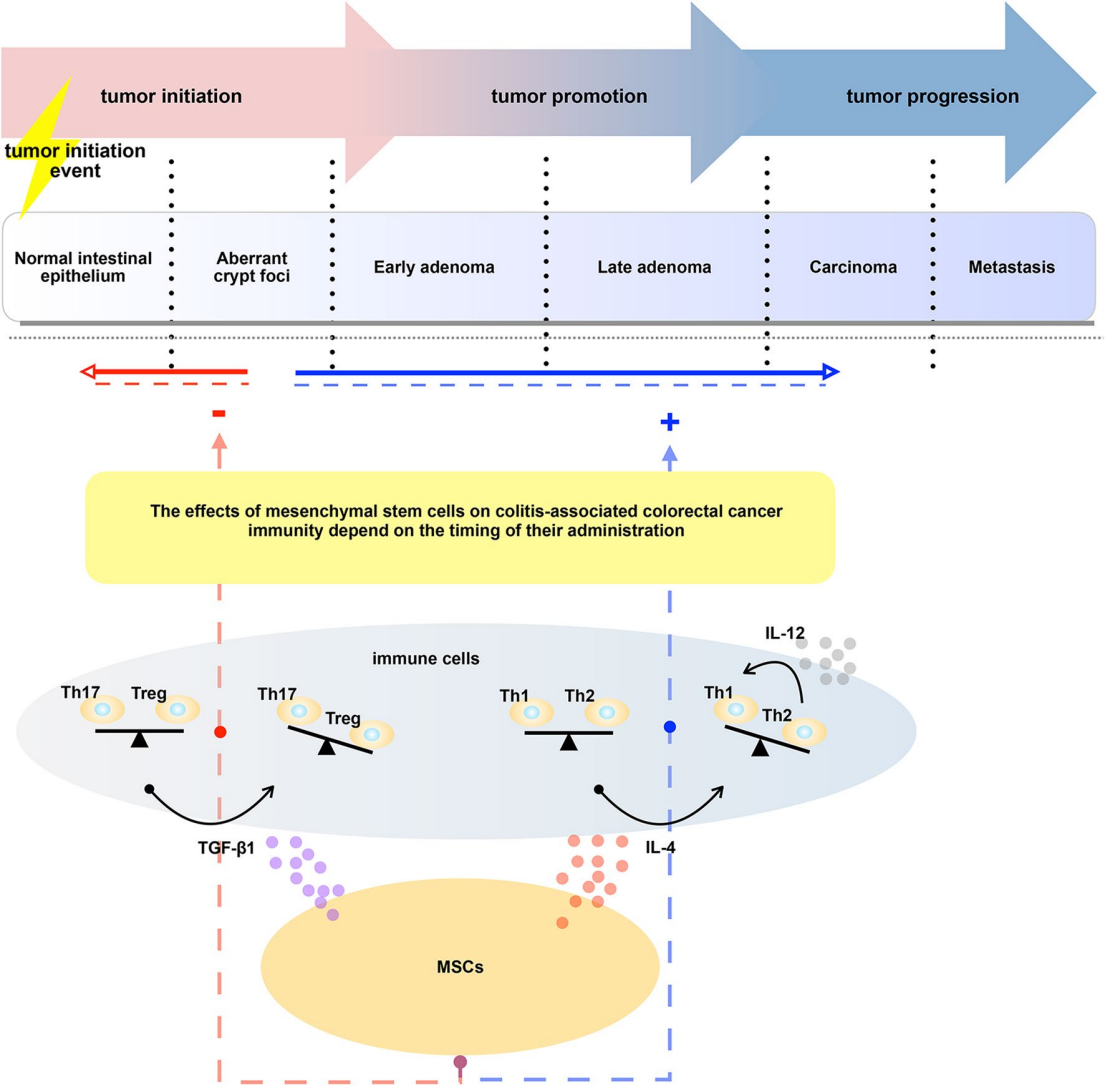
A schematic model that mesenchymal stem cells can curb or promote the progression of colon cancer

This study focuses on the role of MSCs in CAC. The administration of MSCs at different time points alters the course of colon cancer and affects the expression of inflammatory cytokines in colon tissue. MSCs therapy was applied at an early stage, leading to the accumulation of Tregs through TGF-β secretion. When MSCs are administered at an advanced stage, it may induce the shift of Th1/Th2 immune balance to Th2 through IL-4.And the immune balance of Th1/Th2 influenced by MSCs could be reversed by IL-12 (Fig. 7).In the early stages of inflammatory transformation, MSCs can prevent the development of colon cancer, but in the late stages, they can propagate cancer progression.

AOM/DSS-induced mouse colon cancer model is a typical inflammatory cancer transformation model, wherein tumor transformation through repeated induction of inflammation is achieved. At present, immune abnormalities are considered to be an important factor in the pathogenesis of chronic recurrent intestinal inflammatory diseases [29]. MSCs therapy is known to modulate immune responses by inhibiting the proliferation and differentiation of T and B cells [30], interfering with the maturation and normal function of dendritic cells, and regulating other immune cells [31]. However, MSCs themselves do not have the characteristic ability to inhibit the immune response and require to be activated by cytokines such as TGF-β, IFN-γ, and TNF- α to exhibit their corresponding effect [30]. Therefore, we hypothesized that the differential expression of cytokines in the intestinal microenvironment at different stages of inflammatory cancer transformation may have different guiding effects on the activation of transplanted MSCs. The early transplantation of MSCs can reduce the formation of an intestinal tumor, while the transplantation in the later stages can lead to opposite effects.

The impact of MSCs on cancer is controversial [32]. Chen et al. attributed the tumor inhibitory effect of MSCs to the reduction of IL-6 and pSTAT3 signaling in colon cells [33]. Nasuno et al. found that MSCs block cellular DNA damage initiation and induce G1 shutdown in promoter cells via TGF-β signaling [34]. Tsai et al. found that MSCs co-injected in mice with colon cancer promote the occurrence and development of colon cancer [35]. In Kaposi’s sarcoma model, MSCs play an anti-tumor role by inhibiting Akt activity [36]. MSCs can also inhibit the development of human hepatoma cell lines and hepatoma models by involving the WNT signaling pathways [37]. However, there is substantial disagreement on the precise role of MSCs in the origin and growth of tumors, and it is challenging to pinpoint how MSCs directly impact tumors.

In our CAC model, the timing of MSCs application appeared to be a key factor in the contradictory effects of MSCs. Colorectal tumors usually do not develop rapidly in a clinical setting [38]. In AOM/DSS models, the onset of colon tumor formation usually occurred after 5 weeks. We found that MSCs were administered in the early stages of tumor development in all studies investigating the tumor inhibition of MSCs. We speculated that MSCs may promote tumor development once the early tumor initiation phase has passed. Perfecting the exact contribution of MSCs to tumorigenesis at every stage of cancer development is crucial. Previous studies have suggested that the role of MSCs is closely related to tumor-specific environments. The dynamic changes of anti-inflammatory/inflammatory factors are correlated with the dynamic balance of T cell immunity [39]. This dynamic change may be the key to the bidirectional role of MSCs in the transformation of colitis cancer. Our study found that MSCs inhibit tumor development in the early stage and promote tumor development in the late stage of cancers. Inflammatory mediators were detected in intestinal tissue during late MSCs intervention compared to the control group. For example, IL-6 and IL-17 levels were significantly higher in the late MSCs treatment group and lower in the early MSCs treatment group. At the same time, we found more Treg infiltration in the spleen in the early MSCs intervention group, while the Th1/Th2 immune balance shifted to Th2 in the late MSCs intervention group. This suggests that early MSCs intervention may inhibit the progression of CAC mainly by inducing the accumulation of Tregs, while the carcinogenic effect of late MSCs intervention is more likely to be dominated by the dynamic shift of the Th1/Th2 balance to a Th2 phenotype.

TGF-β is an essential cytokine for the differentiation of naïve CD4^+^T cells to CD4^+^CD25^+^Foxp3^+^Treg cells [40]. Treg cells participate in tumor development and progression through tumor immunity [11]. Treg accumulation in cancer has been reported to be associated with poor disease prognosis [11]. However, a substantial positive connection between the density of Foxp3^+^T cell infiltration and improved prognosis and/or survival of CRC models or patients was reported in a review by Ladoire et al. on Foxp3^+^T cell infiltration and prognosis in CRC [41].

The opposite results are not unexpected given that Tregs mediate immune tolerance that promotes tumor growth and inhibits anti-tumor immunity [11]. Tregs can be viewed in this context as an important part of the tumor’s escape from the host immune system [42]; as a result, they may be a sign of poor prognosis and a new target of immunotherapy. CRC develops in the intestinal tract, a special microenvironment rich in a variety of microorganisms. The intestinal mucosal barrier is made up of numerous lymphoid and myeloid cells that are sandwiched between the lamina propria and a single layer of epithelial cells [43]. These T cells and other inflammatory cells are rich in Toll-like receptors (TLRs) which play a crucial role in initiating and maintaining in-situ cell activation [44]. In CAC, Tregs are more likely to inhibit tumor development by reducing damaging inflammation in the early stages of cancer. However, unlike in the early stages, once the tumor is formed, Tregs could be redirected to perform pro-oncogenic rather than anti-oncogenic activities that inhibit the function of tumor antigen-specific effector T cells [45].

Th1 and Th2 are two functional subgroups of CD4^+^ Th cells, which affect the immune response via the secretion of different cytokines [46]. The imbalance of the Th1/Th2 in the body may lead to a variety of conditions including bacterial or viral infections, autoimmune diseases, allergic reactions, and transplant rejection. It is also closely related to the occurrence and development of tumors. A dominant Th1 phenotype confers anti-tumor immunity, whereas a shift to the Th2 phenotype impairs anti-tumor effects. Yamamura et al. found that the dominant Th2 cytokine pattern often occurs in tumor hosts [47]. A shift from Th1 to Th2 has been observed in non-small cell lung cancer [48], choriocarcinoma [49], ovarian cancer [50], glioma [51], kidney cancer [52], colorectal cancer [53, 54], melanoma [55], lymphoma [56], and other types of tumors in the host. IL-4 is one of the main cytokines secreted by Th2 cells and one of the promoters of IL-10 secretion, which can reduce the expression of inflammatory cytokines [57]. Furthermore, it can reduce the expression of MHC antigen-presenting cells, maintain lymphocyte infiltration in the tumor tissue, and form a new type of Th2 infiltrating lymphocytes in the tumor tissue (tumor-infiltrating lymphocytes, TIL) cells, forming TIL–IL-4–IL-10 “Th2 cycle” [58], and could promote the immune escape of tumors [59]. These results provide us with some new ideas for immunotherapy. At the stage of tumor formation, our immunotherapy can mediate the reversal of Th2 to Th1 cells. Gao et al. found taht MSCs produce IL-12 to reduce the growth of renal cell carcinoma of mouse and enhance the tumor-bearing mouse survival [60]. IL-12 favours the differentiation of Th1 cells and forms a link between innate resistance and adaptive immunity [61].We reversed this balance toward Th1 by treating primary T cells with Th1-type fine-related cytokine IL-12 [62]. We suggest that activation of Th1 cells in vivo, such as by anti-Th2 cytokine antibodies, or through active immunity, may be beneficial. If the tumor-host and tumor itself can be promoted to reverse from Th2 type to Th1 type, it will be very beneficial to anti-tumor immunity mainly based on cellular immunity and will be of great significance in conjunction with surgery, radiotherapy, and chemotherapy in preventing tumor recurrence and metastasis as well as improving long-term survival rates.

This research found that MSCs can prevent or promote the progression of colon cancer based on their timing of administration. It provides a basis for the therapeutic schedule of MSCs therapeutics in oncotherapy. One of a key issues in the applications of MSCs therapeutics is the timing. In CAC progression in human, the key to distinguish the early or late stage of MSCs application is whether there is malignant tumor in the intestine. IBD-related CAC evolved from “normal → low atypical hyperplasia → high atypical hyperplasia → adenocarcinoma” [63]. The safety period of MSC application in tumor may be between “normal state-mild atypical hyperplasia” of digestive tract. When moderate and severe dysplasia has occurred, MSCs should be used cautiously.

We found that the accumulation of cancer cells may be the key to altering the microenvironment and potentially reversing the effects of MSCs. Under this hypothesis, the precise mechanism of MSCs on the microenvironment remains to be further explored. In addition, key molecules that can reverse the effects of MSCs should be identified to serve as important reference factors for future MSC therapy.

## Conclusion

MSCs can prevent the progression of colon cancer by inducing Treg accumulation via TGF-β at the early stage of inflammatory transformation but promote the progression of colon cancer by inducing a shift in Th1/Th2 immune balance to Th2 through IL-4 secretion at the late stage (Fig. 7). This study provides a basis for the therapeutic schedule and timing of MSCs therapeutics for improving prognosis and survival outcomes following tumor therapy.

## Supplementary Information

**Additional file 1.** The specific primers’ sequences for RNA amplification.

**Additional file 2.** Original blots of Western Blot analysis. The figure legend of this file is the same as the legend of the corresponding figure in the main text.

## Data Availability

The datasets used and analyzed in the current study are available from the corresponding author upon reasonable request.

## Abbreviation

MSCs: Mesenchymal stem cells
BM-MSCs: Bone marrow-derived MSCs
CAC: Colitis-associated colon cancer
AOM: Azoxymethane
DSS: Dextran sulfate sodium
TGF-β: Transforming growth factor-β
Th: Helper T cell
CRC: Colorectal cancer
TME: Tumor microenvironment
IFN-γ: Interferon-γ
IL-: Interleukin-
TNF-α: Tumor necrosis factor-α
Tregs: Regulatory T cells
IBD: Inflammatory bowel disease
RT-qPCR: Real-time quantitative PCR
WB: Western blot
LPL: Isolation of splenocytes and lamina propria lymphocytes
