# Supplementary figures and images for: Mesenchymal stem cells can prevent or promote the progression of colon cancer based on their timing of administration
Source: J Transl Med. 2023 Mar 28;21:227. doi: 10.1186/s12967-023-04028-3 (PMC10045613; doi:10.1186/s12967-023-04028-3)

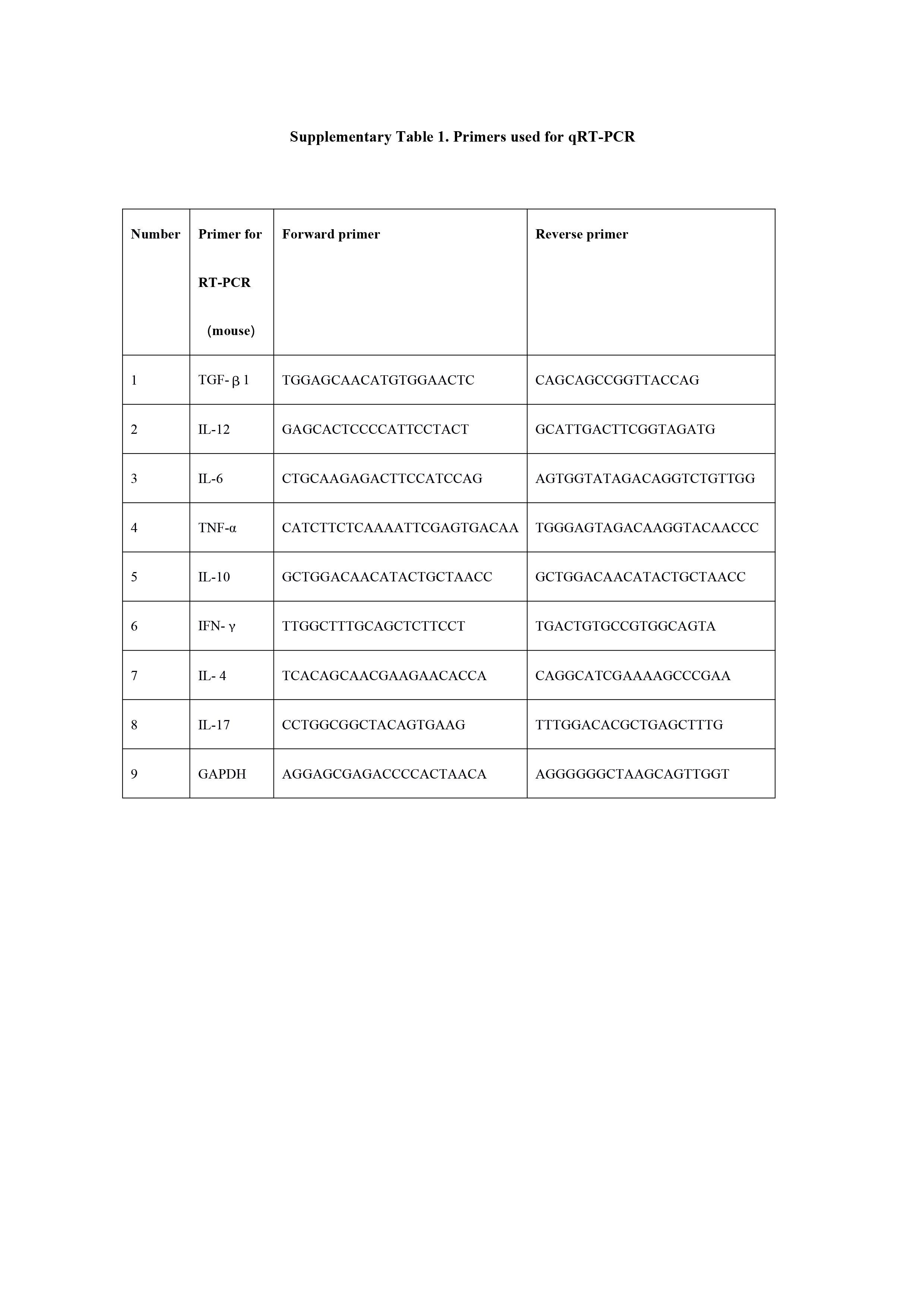

Supplement: Supplementary file 1 — Additional file 1. The specific primers’ sequences for RNA amplification. [file 12967_2023_4028_MOESM1_ESM.tif]

Fig. S1

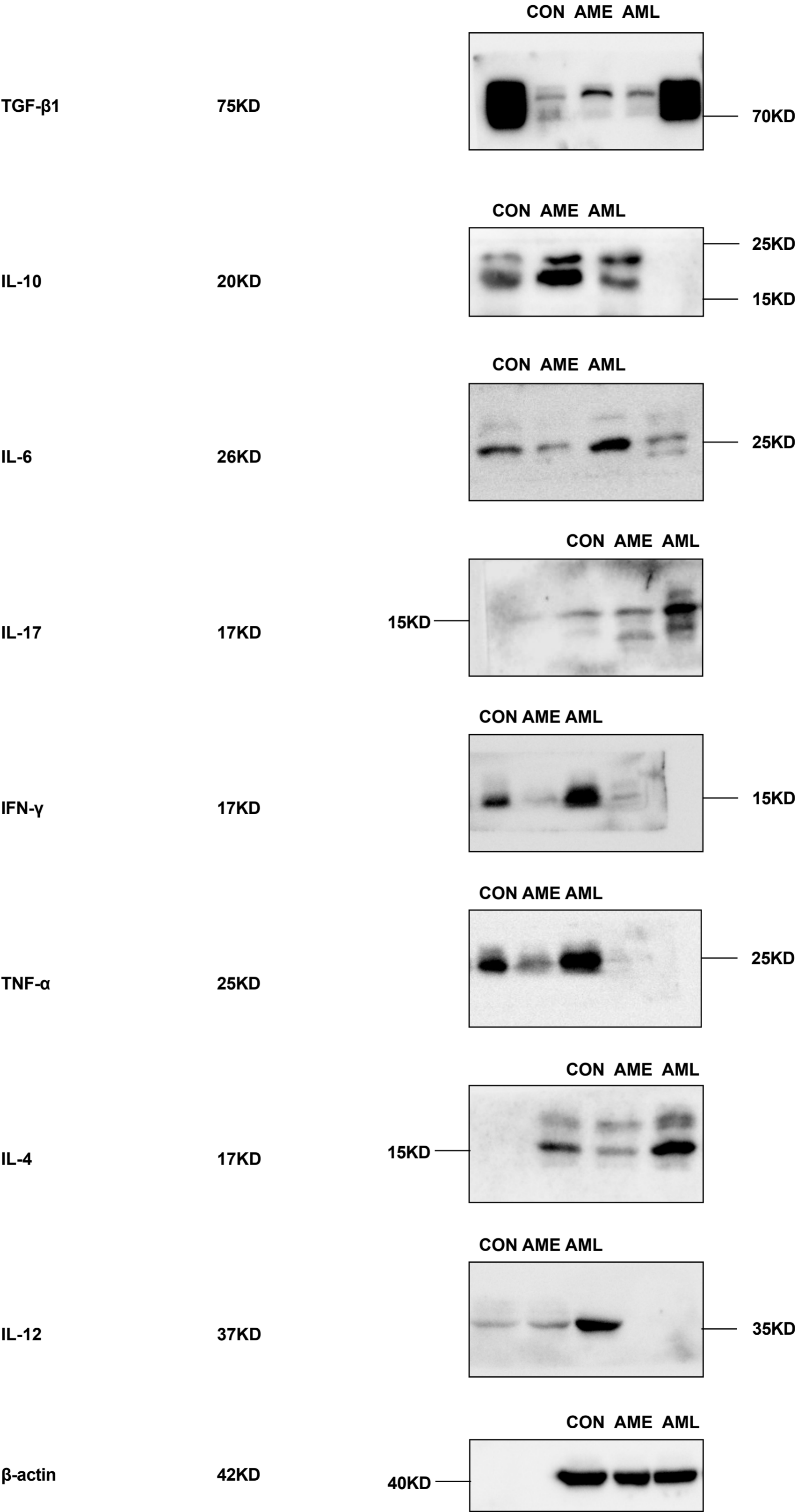

Fig. S2

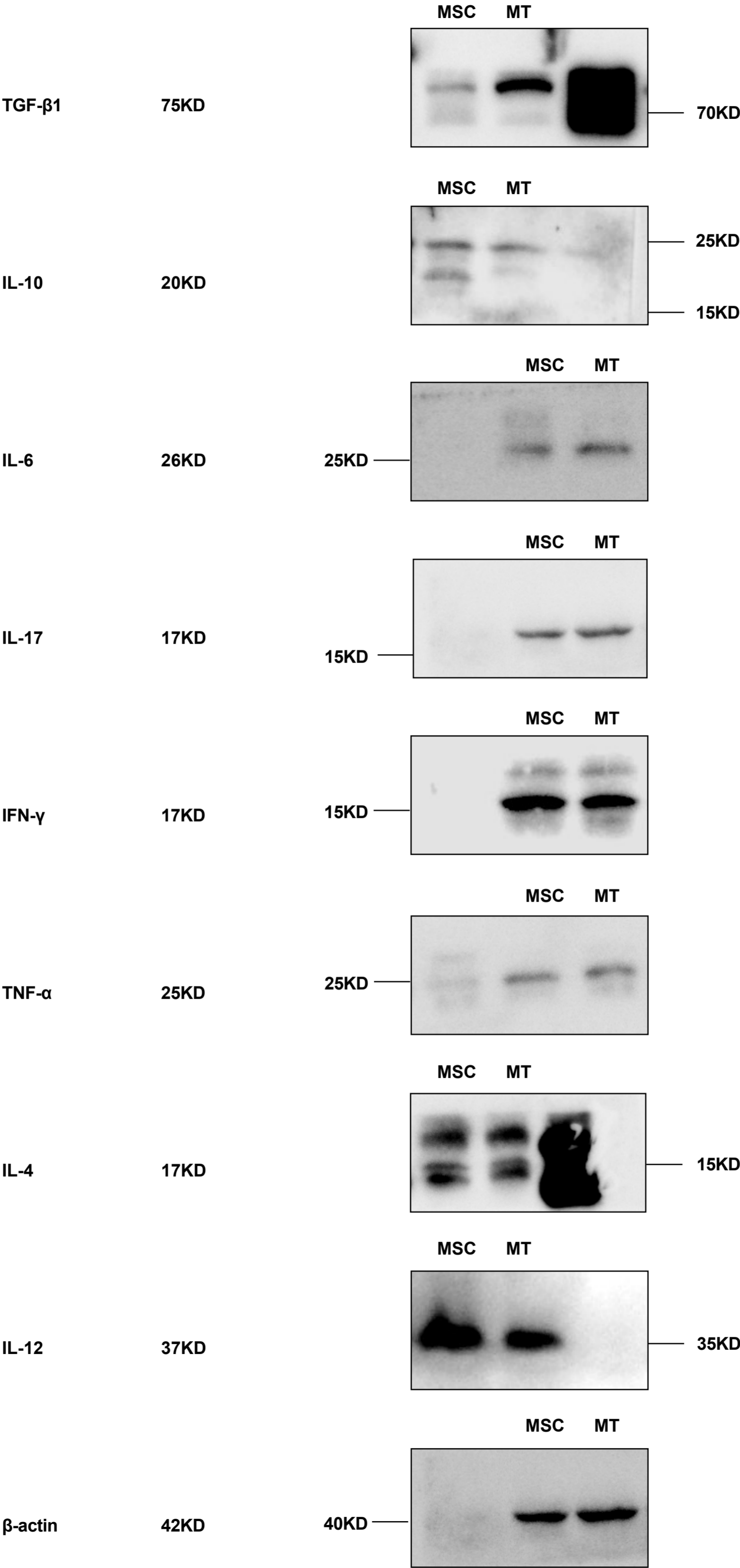

Fig. S3

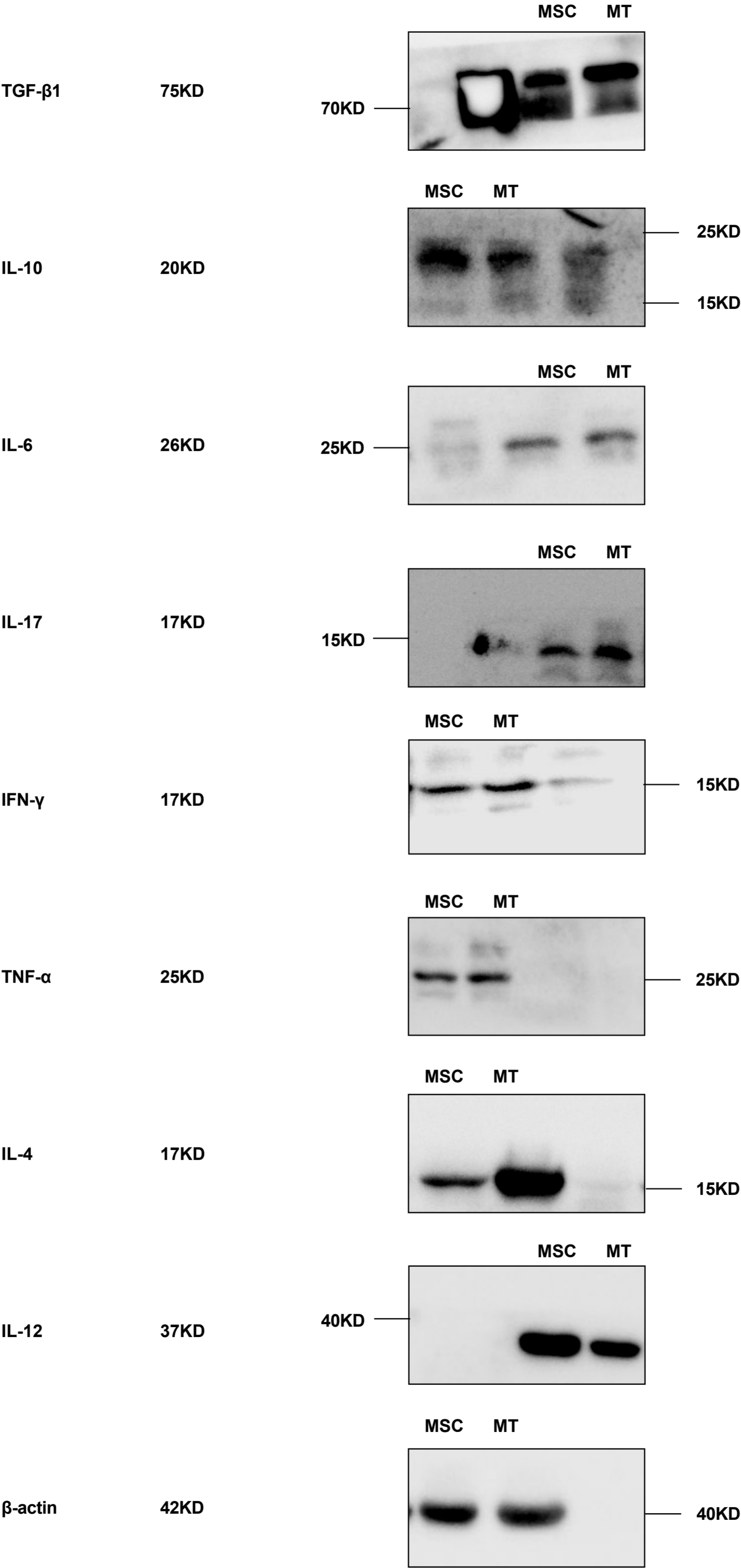

Supplement: Supplementary file 2 — Additional file 2. Original blots of Western Blot analysis. The figure legend of this file is the same as the legend of the corresponding figure in the main text. [file 12967_2023_4028_MOESM2_ESM.pdf]
